# *catena*-Poly[{μ_3_-3,3′-[(1,7-dioxa-4,10-di­aza­cyclo­dodecane-4,10-di­yl)bis­(methyl­ene)]dibenzoato}cobalt(II)]

**DOI:** 10.1107/S1600536813032832

**Published:** 2013-12-18

**Authors:** Liang Liao, Conrad W. Ingram, John Bacsa, Cass Parker

**Affiliations:** a Center for Functional Nanoscale Materials, Department of Chemistry, Clark Atlanta University, 223 James P. Brawley Drive, Atlanta, GA 30314, USA; b X-ray Crystallography Center, Emory University, Atlanta, GA 30322, USA

## Abstract

The title compound, [Co(C_24_H_28_N_2_O_6_)]_*n*_, crystallizes as infinite chains related to one another by inversion centers, giving a centrosymmetric coordination polymer. The Co^II^ ion, situated on a twofold rotation axis, forms a complex with the crown-4 moiety of the 3,3′-[(1,7-dioxa-4,10-di­aza­cyclo­do­decane-4,10-di­yl)bis­(meth­ylene)]dibenzoate anion. The dis­torted octahedral coordination sphere of the Co^II^ ion is completed by two carboxyl­ate O atoms from two bridging intra-chain ligands. Metallomacrocyclic rings of 16 atoms are present, with each ring containing two Co^II^ ions and 14 atoms from the bridging ligands. These units repeat as infinite zigzag chains along [101].

## Related literature   

For the structures of coordination polymers (CPs) or compounds with metal-organic frameworks including one-dimensional CPs or MOFs, see: Du *et al.* (2013 ▶); Ingram *et al.* (2012 ▶, 2013 ▶); Janiak (2013 ▶); Leong & Vittal (2011 ▶).
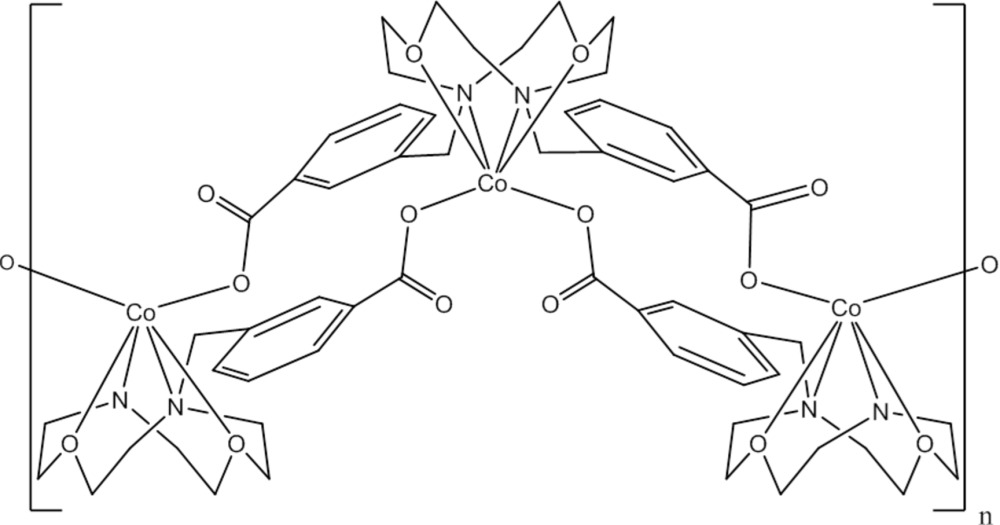


## Experimental   

### 

#### Crystal data  

[Co(C_24_H_28_N_2_O_6_)]
*M* *_r_* = 499.41
Monoclinic, 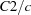

*a* = 20.626 (2) Å
*b* = 8.9778 (10) Å
*c* = 13.9263 (16) Å
β = 127.051 (1)°
*V* = 2058.2 (4) Å^3^
*Z* = 4
Mo *K*α radiation
μ = 0.88 mm^−1^
*T* = 173 K
0.40 × 0.14 × 0.14 mm

#### Data collection  

Bruker APEXII CCD diffractometer
Absorption correction: multi-scan (*SADABS*; Bruker, 2012) ▶ *T*_min_ = 0.606, *T*_max_ = 0.746
3614 measured reflections
2930 independent reflections
2290 reflections with *I* > 2σ(*I*)
*R*_int_ = 0.018

#### Refinement  

*R*[*F* ^2^ > 2σ(*F* ^2^)] = 0.049
*wR*(*F* ^2^) = 0.125
*S* = 1.02
2930 reflections
150 parameters
H-atom parameters constrained
Δρ_max_ = 0.80 e Å^−3^
Δρ_min_ = −0.47 e Å^−3^

### 

Data collection: *APEX2* (Bruker, 2011 ▶); cell refinement: *SAINT* (Bruker, 2009 ▶); data reduction: *SAINT*; program(s) used to solve structure: *SHELXS97* (Sheldrick, 2008 ▶); program(s) used to refine structure: *SHELXL97* (Sheldrick, 2008 ▶); molecular graphics: *OLEX2* (Dolomanov *et al.*, 2009 ▶); software used to prepare material for publication: *OLEX2*.

## Supplementary Material

Crystal structure: contains datablock(s) I. DOI: 10.1107/S1600536813032832/gg2131sup1.cif

Structure factors: contains datablock(s) I. DOI: 10.1107/S1600536813032832/gg2131Isup2.hkl

Supporting information file. DOI: 10.1107/S1600536813032832/gg2131Isup3.cdx

Additional supporting information:  crystallographic information; 3D view; checkCIF report

## Figures and Tables

**Fig. 1.**
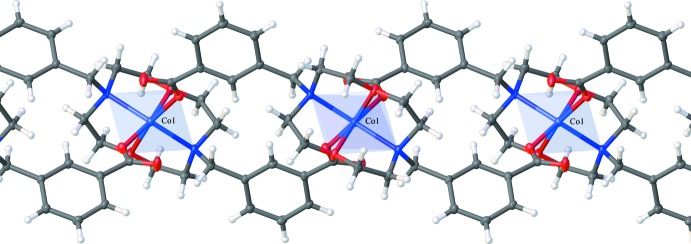
A view of a portion of one of the chains of (I). Non-H atoms are represented by ellipsoids at the 50% probability level. Sixteen membered metallomacrocycle rings can be identified from this figure.

## supplementary crystallographic information

### 1. Comment

The title compound is the one of a series of coordination polymers prepared from the anionic ligand LH2, 3,3'-((1,7-dioxa-4,10-diazacyclododecane-4,10-diyl)bis(methylene))dibenzoate. This ligand shows unusual adaptability in that it displays two complexation modes on binding to metals. The ligand attaches to the metal *via* two oxygen and two nitrogen atoms (forming a crown complex). The crown forms four bonds to the metal, while an ideal coordination number for a Co^II^ ion is 6. Thus vacant coordination sites suitable for coordination by the carboxylate groups exist. The carboxylate ions behave as monodentate bridging ligands and the entire ligand is hexadentate. The Co^II^ atom is moved out of the best plane of the crown since this arrangement is better for forming optimal bonds to the ligand. This new compound is novel in that, although the ligands bridge the metal atoms forming one-dimensional chains, the metal atoms are positioned in the center of the organic linker. Topologically, the Co^II^ atoms and the ligands forms nodes in the network rather than the metal atoms only.

The title compound is synthesized from the ligand LH2, 3,3'-((1,7-dioxa-4,10-diazacyclododecane-4,10-diyl)bis(methylene)) dibenzoic acid. The metal atoms are positioned in the center of the organic linker. The asymmetric unit of the compound contains a Co^II^ ion and a deprotonated ligand *L* with formula C_24_H_28_N_2_O_6_Co. The Co^II^ ion is 6-coordinate in a distorted octahedral geometry being bound to two N atoms and two O atoms of the crown (1,7-diaza-12-crown-4) and two carboxylic O atoms, one from each of two additional intra-chain ligands (Figure 1s). The Co1—O1, Co1—O3 and Co1—N1 bond lengths are 1.9886 (16), 2.2399 (16) and 2.2213 (17) Å, respectively. The O1—Co1—O1 angle is 104.15 (9)°. The shortest distance between two neighboring Co^II^ ions along a chain is 9.046 (1) Å. The Co^II^ ion of the Co(crown-4)2+ unit is located on a 2-fold rotation axis. The symmetry independent atoms consist of one half of the ligand with the rotation axis generating the second half of the ligand at the Co atom. Bond circuits consisting of sixteen-membered metallomacrocycle rings can be identified in the structure. Each ring contains two Co^II^ ions and fourteen non-H atoms of the ligand. Each Co^II^ ion is a node for three ligands and two connected macrocycle rings. The pair of benzene moieties within a metallomacrocycle ring are remarkably co-planar (the two rings are in the same plane within experimental error). The dihedral angle between this plane and the plane of the next two nearest phenyl rings along the 1-D chain is 68.79 (5)°. Repetition of these units creates a 1-D polymer network with an infinite number of these rings.

### 2. Experimental

The title compound was synthesized in an autoclave by mixing the ligand, 3,3'-((1,7-dioxa-4,10-diazacyclododecane-4,10-diyl)bis(methylene))dibenzoic acid, LH_2_ (4x10^-5^ mol), (Ingram *et al.* (2012), (2013)) Co(NO_3_)_2_·6H_2_O (1.2x10^-4^ mol, 35.8 mg), H_2_O (12 ml) and pyridine (4x10^-2^ ml). The mixture was heated at 130 °C in an autoclave for 7 days and then cooled to ambient temperature. Red crystals were collected and washed with H_2_O by filtration. Elem. anal. calcd. C_24_H_28_N_2_O_6_Co %: C, 57.72; H, 5.65; N, 5.61; Found: C, 57.79; H, 5.74; N, 5.46.

### 3. Refinement

Refinement Refinement of F2 against ALL reflections. The weighted *R*-factor *wR* and goodness of fit S are based on F2, conventional *R*-factors *R* are based on F, with F set to zero for negative F2. The threshold expression of F2 > 2sigma(F2) is used only for calculating *R*-factors(gt), *etc* and is not relevant to the choice of reflections for refinement. *R*-factors based on F2 are statistically about twice as large as those based on F and R– factors based on ALL data will be even larger. Computing details Data collection: *APEX2* (Bruker, 2011); cell refinement: *SAINT* (Bruker, 2009); data reduction: *SAINT* (Bruker, 2009);program(*s*) used to solve structure: *SHELXS97* (Sheldrick, 2008); program(*s*) used to refine structure: *SHELXL97* (Sheldrick, 2008); molecular graphics: OLEX2 (Dolomanov *et al.*, 2009); software used to prepare material for publication: OLEX2 (Dolomanov *et al.*, 2009).

### Crystal data

**Table:**

[Co(C_24_H_28_N_2_O_6_)]	*F*(000) = 1044
*M**_r_* = 499.41	*D*_x_ = 1.612 Mg m^−^^3^
Monoclinic, *C*2/*c*	Mo *K*α radiation, λ = 0.71073 Å
*a* = 20.626 (2) Å	Cell parameters from 4901 reflections
*b* = 8.9778 (10) Å	θ = 2.5–31.0°
*c* = 13.9263 (16) Å	µ = 0.88 mm^−^^1^
β = 127.051 (1)°	*T* = 173 K
*V* = 2058.2 (4) Å^3^	Needle, red
*Z* = 4	0.40 × 0.14 × 0.14 mm

### Data collection

**Table:**

Bruker D8 diffractometer with a APEXII detector	2930 independent reflections
Radiation source: fine-focus sealed tube	2290 reflections with *I* > 2σ(*I*)
Graphite monochromator	*R*_int_ = 0.018
Detector resolution: 512 pixels mm^-1^	θ_max_ = 31.2°, θ_min_ = 2.6°
φ and ω scans with a narrow frame width	*h* = −28→18
Absorption correction: multi-scan (*SADABS*; Bruker, 2012)	*k* = −12→4
*T*_min_ = 0.606, *T*_max_ = 0.746	*l* = −20→19
3614 measured reflections	

### Refinement

**Table:**

Refinement on *F*^2^	Primary atom site location: iterative
Least-squares matrix: full	Secondary atom site location: difference Fourier map
*R*[*F*^2^ > 2σ(*F*^2^)] = 0.049	Hydrogen site location: difference Fourier map
*wR*(*F*^2^) = 0.125	H-atom parameters constrained
*S* = 1.02	*w* = 1/[σ^2^(*F*_o_^2^) + (0.073*P*)^2^] where *P* = (*F*_o_^2^ + 2*F*_c_^2^)/3
2930 reflections	(Δ/σ)_max_ < 0.001
150 parameters	Δρ_max_ = 0.80 e Å^−^^3^
0 restraints	Δρ_min_ = −0.47 e Å^−^^3^

### Special details

**Table:**

Experimental. Absorption correction: SADABS-2012/1 (Bruker,2012) was used for absorption correction. wR2(int) was 0.0566 before and 0.0407 after correction. The Ratio of minimum to maximum transmission is 0.8118. The λ/2 correction factor is 0.0015.
Geometry. All e.s.d.'s (except the e.s.d. in the dihedral angle between two l.s. planes) are estimated using the full covariance matrix. The cell e.s.d.'s are taken into account individually in the estimation of e.s.d.'s in distances, angles and torsion angles; correlations between e.s.d.'s in cell parameters are only used when they are defined by crystal symmetry. An approximate (isotropic) treatment of cell e.s.d.'s is used for estimating e.s.d.'s involving l.s. planes.

### Fractional atomic coordinates and isotropic or equivalent isotropic displacement parameters (Å^2^)

**Table:**

	*x*	*y*	*z*	*U*_iso_*/*U*_eq_	
H3	0.2131	−0.1861	0.3961	0.018*	
H5	0.3214	0.0551	0.2858	0.021*	
H6	0.1962	0.0922	0.1030	0.020*	
H7	0.0799	−0.0063	0.0656	0.017*	
H8a	0.3537	−0.1744	0.5242	0.018*	
H8b	0.3996	−0.1315	0.4708	0.018*	
H9a	0.3126	0.0166	0.5936	0.022*	
H9b	0.2955	0.1475	0.5068	0.022*	
H10a	0.3343	0.2306	0.6986	0.025*	
H10b	0.3890	0.2984	0.6656	0.025*	
H11a	0.4755	0.2883	0.9128	0.023*	
H11b	0.5171	0.3117	0.8492	0.023*	
H12a	0.3833	0.2132	0.4673	0.022*	
H12b	0.4423	0.0940	0.4768	0.022*	
C1	0.06017 (14)	−0.1928 (2)	0.19467 (19)	0.0158 (4)	
C2	0.13474 (13)	−0.1115 (2)	0.22645 (18)	0.0134 (4)	
C3	0.20986 (14)	−0.1300 (2)	0.33718 (19)	0.0148 (4)	
C4	0.28076 (14)	−0.0671 (2)	0.36296 (18)	0.0139 (4)	
C5	0.27494 (14)	0.0150 (3)	0.27221 (19)	0.0174 (5)	
C6	0.19972 (15)	0.0363 (2)	0.16222 (19)	0.0171 (4)	
C7	0.12995 (14)	−0.0239 (2)	0.13905 (19)	0.0143 (4)	
C8	0.36136 (14)	−0.0955 (2)	0.48429 (19)	0.0153 (4)	
C9	0.33771 (14)	0.0978 (3)	0.5810 (2)	0.0185 (5)	
C10	0.37353 (14)	0.2069 (3)	0.6840 (2)	0.0205 (5)	
C11	0.50015 (14)	0.2376 (2)	0.8806 (2)	0.0194 (5)	
C12	0.42768 (14)	0.1462 (2)	0.52219 (19)	0.0184 (5)	
N1	0.39880 (11)	0.03569 (18)	0.56797 (16)	0.0130 (4)	
O1	0.07535 (10)	−0.31870 (17)	0.24829 (14)	0.0177 (3)	
O2	−0.00750 (10)	−0.1403 (2)	0.11880 (15)	0.0266 (4)	
O3	0.44337 (10)	0.13620 (18)	0.78732 (13)	0.0184 (3)	
Co1	0.0000	−0.45483 (4)	0.2500	0.01262 (13)	

### Atomic displacement parameters (Å^2^)

**Table:**

	*U*^11^	*U*^22^	*U*^33^	*U*^12^	*U*^13^	*U*^23^
C1	0.0172 (12)	0.0174 (10)	0.0141 (9)	−0.0039 (8)	0.0102 (9)	−0.0025 (8)
C2	0.0121 (11)	0.0108 (9)	0.0152 (9)	−0.0006 (7)	0.0070 (8)	−0.0018 (7)
C3	0.0175 (11)	0.0117 (9)	0.0141 (9)	0.0000 (8)	0.0090 (9)	0.0014 (7)
C4	0.0128 (11)	0.0142 (10)	0.0121 (9)	0.0002 (7)	0.0062 (8)	−0.0003 (7)
C5	0.0153 (12)	0.0193 (11)	0.0161 (10)	−0.0031 (8)	0.0086 (9)	−0.0003 (8)
C6	0.0205 (12)	0.0151 (10)	0.0148 (9)	−0.0033 (8)	0.0103 (9)	0.0013 (8)
C7	0.0138 (11)	0.0138 (10)	0.0120 (9)	0.0012 (8)	0.0060 (8)	0.0005 (7)
C8	0.0132 (11)	0.0135 (9)	0.0154 (9)	0.0002 (8)	0.0066 (9)	−0.0007 (7)
C9	0.0120 (11)	0.0204 (11)	0.0165 (10)	0.0034 (8)	0.0052 (9)	0.0006 (8)
C10	0.0167 (12)	0.0202 (11)	0.0181 (10)	0.0061 (9)	0.0071 (9)	0.0003 (8)
C11	0.0162 (12)	0.0169 (11)	0.0178 (10)	0.0027 (8)	0.0064 (9)	−0.0049 (8)
C12	0.0178 (12)	0.0175 (10)	0.0153 (9)	−0.0032 (8)	0.0075 (9)	0.0035 (8)
N1	0.0104 (9)	0.0122 (8)	0.0139 (8)	−0.0011 (6)	0.0059 (7)	−0.0004 (6)
O1	0.0156 (8)	0.0148 (7)	0.0219 (8)	0.0001 (6)	0.0110 (7)	0.0024 (6)
O2	0.0132 (9)	0.0302 (10)	0.0244 (8)	−0.0007 (7)	0.0049 (7)	0.0103 (7)
O3	0.0140 (8)	0.0165 (7)	0.0152 (7)	0.0027 (6)	0.0037 (6)	−0.0016 (6)
Co1	0.0105 (2)	0.0114 (2)	0.0141 (2)	0.000	0.00639 (17)	0.000

### Geometric parameters (Å, º)

**Table:**

C1—C2	1.508 (3)	C11—H11b	0.9700
C2—C3	1.388 (3)	C11—C12^i^	1.515 (3)
C2—C7	1.402 (3)	C12—H12a	0.9700
C3—H3	0.9300	C12—H12b	0.9700
C4—C3	1.398 (3)	C12—C11^i^	1.515 (3)
C4—C8	1.516 (3)	N1—C8	1.502 (3)
C5—H5	0.9300	N1—C9	1.486 (3)
C5—C4	1.404 (3)	N1—C12	1.484 (3)
C6—H6	0.9300	N1—Co1^ii^	2.2212 (17)
C6—C5	1.388 (3)	O1—C1	1.285 (3)
C7—H7	0.9300	O2—C1	1.229 (3)
C7—C6	1.381 (3)	O3—C10	1.432 (3)
C8—H8a	0.9700	O3—C11	1.433 (3)
C8—H8b	0.9700	O3—Co1^ii^	2.2400 (16)
C9—H9a	0.9700	Co1—N1^iii^	2.2213 (17)
C9—H9b	0.9700	Co1—N1^ii^	2.2213 (17)
C9—C10	1.511 (3)	Co1—O1	1.9886 (16)
C10—H10a	0.9700	Co1—O1^iv^	1.9886 (16)
C10—H10b	0.9700	Co1—O3^iii^	2.2399 (16)
C11—H11a	0.9700	Co1—O3^ii^	2.2399 (16)
			
O1—C1—C2	114.0 (2)	O3—C10—C9	106.67 (18)
O2—C1—C2	119.75 (19)	H11a—C11—H11b	108.6
O2—C1—O1	126.2 (2)	C12^i^—C11—H11a	110.3
C3—C2—C1	121.76 (19)	C12^i^—C11—H11b	110.3
C3—C2—C7	118.7 (2)	O3—C11—H11a	110.3
C7—C2—C1	119.40 (19)	O3—C11—H11b	110.3
C2—C3—H3	118.9	O3—C11—C12^i^	107.00 (17)
C2—C3—C4	122.10 (19)	H12a—C12—H12b	107.6
C4—C3—H3	118.9	C11^i^—C12—H12a	108.7
C3—C4—C5	118.2 (2)	C11^i^—C12—H12b	108.7
C3—C4—C8	119.56 (19)	N1—C12—H12a	108.7
C5—C4—C8	122.2 (2)	N1—C12—H12b	108.7
C4—C5—H5	120.1	N1—C12—C11^i^	114.25 (17)
C6—C5—H5	120.1	C8—N1—Co1^ii^	108.63 (12)
C6—C5—C4	119.9 (2)	C9—N1—C8	108.17 (17)
C5—C6—H6	119.4	C9—N1—Co1^ii^	105.43 (12)
C7—C6—H6	119.4	C12—N1—C8	110.02 (17)
C7—C6—C5	121.2 (2)	C12—N1—C9	113.03 (17)
C2—C7—H7	120.1	C12—N1—Co1^ii^	111.36 (13)
C6—C7—H7	120.1	C1—O1—Co1	128.97 (15)
C6—C7—C2	119.9 (2)	C10—O3—C11	114.03 (17)
H8a—C8—H8b	107.4	C10—O3—Co1^ii^	116.01 (13)
C4—C8—H8a	108.3	C11—O3—Co1^ii^	114.60 (13)
C4—C8—H8b	108.3	N1^iii^—Co1—N1^ii^	141.85 (9)
N1—C8—H8a	108.3	N1^iii^—Co1—O3^iii^	76.37 (6)
N1—C8—H8b	108.3	N1^ii^—Co1—O3^iii^	76.14 (6)
N1—C8—C4	116.03 (17)	N1^ii^—Co1—O3^ii^	76.37 (6)
H9a—C9—H9b	107.8	N1^iii^—Co1—O3^ii^	76.14 (6)
C10—C9—H9a	108.9	O1—Co1—N1^ii^	90.61 (7)
C10—C9—H9b	108.9	O1^iv^—Co1—N1^ii^	113.02 (7)
N1—C9—H9a	108.9	O1^iv^—Co1—N1^iii^	90.61 (7)
N1—C9—H9b	108.9	O1—Co1—N1^iii^	113.02 (7)
N1—C9—C10	113.18 (19)	O1^iv^—Co1—O1	104.15 (9)
H10b—C10—H10a	108.6	O1—Co1—O3^ii^	85.61 (6)
C9—C10—H10a	110.4	O1^iv^—Co1—O3^iii^	85.61 (6)
C9—C10—H10b	110.4	O1—Co1—O3^iii^	165.96 (6)
O3—C10—H10a	110.4	O1^iv^—Co1—O3^ii^	165.96 (6)
O3—C10—H10b	110.4	O3^iii^—Co1—O3^ii^	86.74 (9)
			
C1—C2—C3—C4	173.4 (2)	C10—O3—C11—C12^i^	−175.92 (19)
C1—C2—C7—C6	−172.2 (2)	C11—O3—C10—C9	159.56 (19)
C2—C7—C6—C5	−1.7 (3)	C12—N1—C8—C4	−70.1 (2)
C3—C2—C7—C6	2.9 (3)	C12—N1—C9—C10	−71.2 (2)
C3—C4—C8—N1	−110.1 (2)	N1—C9—C10—O3	−49.7 (3)
C5—C4—C3—C2	−1.1 (3)	O1—C1—C2—C3	−28.4 (3)
C5—C4—C8—N1	73.0 (3)	O1—C1—C2—C7	146.5 (2)
C6—C5—C4—C3	2.4 (3)	O2—C1—C2—C3	155.1 (2)
C6—C5—C4—C8	179.3 (2)	O2—C1—C2—C7	−30.0 (3)
C7—C2—C3—C4	−1.5 (3)	Co1^ii^—N1—C8—C4	167.72 (15)
C7—C6—C5—C4	−1.0 (3)	Co1^ii^—N1—C9—C10	50.7 (2)
C8—C4—C3—C2	−178.10 (19)	Co1^ii^—N1—C12—C11^i^	−30.1 (2)
C8—N1—C9—C10	166.73 (18)	Co1—O1—C1—C2	172.68 (13)
C8—N1—C12—C11^i^	−150.6 (2)	Co1—O1—C1—O2	−11.1 (3)
C9—N1—C8—C4	53.8 (2)	Co1^ii^—O3—C10—C9	23.1 (2)
C9—N1—C12—C11^i^	88.3 (2)	Co1^ii^—O3—C11—C12^i^	−38.8 (2)

Symmetry codes: (i) −*x*+1, *y*, −*z*+3/2; (ii) −*x*+1/2, −*y*−1/2, −*z*+1; (iii) *x*−1/2, −*y*−1/2, *z*−1/2; (iv) −*x*, *y*, −*z*+1/2.
